# Probe set algorithms: is there a rational best bet?

**DOI:** 10.1186/1471-2105-7-395

**Published:** 2006-08-30

**Authors:** Jinwook Seo, Eric P Hoffman

**Affiliations:** 1 Research Center for Genetic Medicine, Children's National Medical Center, 111 Michigan Ave NW, Washington DC 20010, USA

## Abstract

Affymetrix microarrays have become a standard experimental platform for studies of mRNA expression profiling. Their success is due, in part, to the multiple oligonucleotide features (probes) against each transcript (probe set). This multiple testing allows for more robust background assessments and gene expression measures, and has permitted the development of many computational methods to translate image data into a single normalized "signal" for mRNA transcript abundance. There are now many probe set algorithms that have been developed, with a gradual movement away from chip-by-chip methods (MAS5), to project-based model-fitting methods (dCHIP, RMA, others). Data interpretation is often profoundly changed by choice of algorithm, with disoriented biologists questioning what the "accurate" interpretation of their experiment is. Here, we summarize the debate concerning probe set algorithms. We provide examples of how changes in mismatch weight, normalizations, and construction of expression ratios each dramatically change data interpretation. All interpretations can be considered as computationally appropriate, but with varying biological credibility. We also illustrate the performance of two new hybrid algorithms (PLIER, GC-RMA) relative to more traditional algorithms (dCHIP, MAS5, Probe Profiler PCA, RMA) using an interactive power analysis tool. PLIER appears superior to other algorithms in avoiding false positives with poorly performing probe sets. Based on our interpretation of the literature, and examples presented here, we suggest that the variability in performance of probe set algorithms is more dependent upon assumptions regarding "background", than on calculations of "signal". We argue that "background" is an enormously complex variable that can only be vaguely quantified, and thus the "best" probe set algorithm will vary from project to project.

## Mini-intro to the Affymetrix array

Current Affymetrix microarrays [1] use chemical synthesis of 25 mer oligonucleotides in 8 μm^2 ^features on glass slides, resulting in a density of approximately 1 million oligonucleotides per 1.3 cm^2 ^microarray. The impressive density of oligonucleotides provides adequate space on the chip ("real estate") for use of multiple probes per mRNA transcript. Thus, each transcript is queried by a probe set (typically 22 oligonucleotides on current arrays). The assessment of the amount of a specific mRNA in a solution from a tissue or cell is determined by the amount of hybridization to the different oligo features for that mRNA. While "hybridization" is a highly sensitive and specific assay, it is not perfectly sensitive or perfectly specific. Thus, it is critical to provide measures of non-specific hybridization and probe binding (background), and one method is to print mismatch probes, where a single base mutation in the center of each 25 mer probe should disrupt most "specific" hybridization. Current expression profiling microarrays use 11 perfect match and mismatch probe pairs for each probe set, although there may be many probe sets per transcript unit.

**Figure 1:**
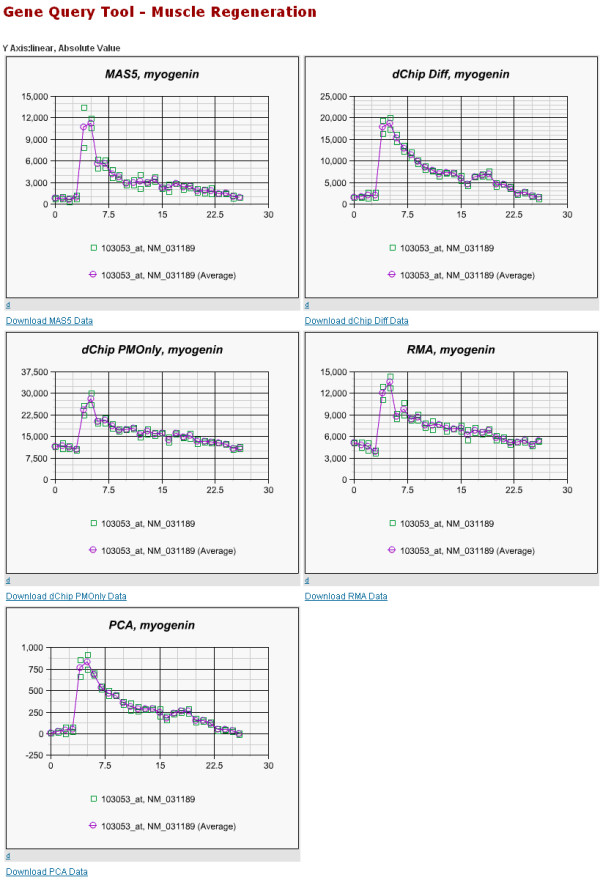
**Effects of probe set algorithms on absolute expression values and variance**. Shown is the same 54 microarray data set from a muscle regeneration 27 time point temporal series [25], analyzed by five different probe set algorithms. While all probe set algorithms show the same transcriptional induction of this transcript at the day 3.0 time point (expression pattern), the absolute expression levels both at baseline (time 0), and peak expression (day 3.0) computed by the probe set algorithms vary significantly. For example, at baseline, MAS5, dCHIP Difference model, and PCA all show expression near background levels (0), while dCHIP perfect match and RMA show baseline values at 5,000–10,000 units. All graphs are output of the PEPR public access tool [26].

**Figure 2:**
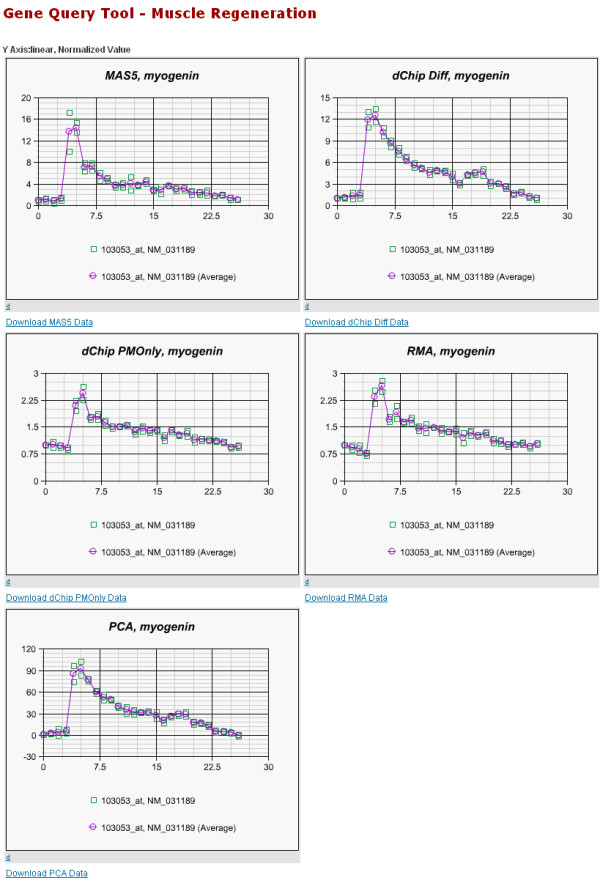
**Effects of probe set algorithms on fold-change calculations**. Shown is the same data set in Figure 1, now normalized to time 0, with the Y axis showing fold-change. The calculated fold change from day 0 (baseline) to peak transcript induction at time point 3.0 days varies considerably from algorithm to algorithm. For example, PCA algorithm shows a 90-fold induction compared to baseline, while RMA and dCHIP show only 2.5-fold induction.

The multiple measurements of hybridization for each mRNA allow a more robust measure of expression, as "signal" is derived from many assessments. However, the multiple measures has led to considerable debate with regards to the best method of integrating the 11 perfect match and 11 paired mismatch hybridization intensities into an ensembled "signal" for each gene. There are many calculations intrinsic to the probe set algorithms that derive gene expression signal, including varied penalties for the mismatch signal, different intra-probe set normalizations, and normalizations between microarrays. Recent publications have reviewed the specific calculations used by different probe set algorithms [2,3]. To illustrate the effects of choice of probe set algorithm on data interpretation, a 27 time point series data was analyzed with 5 different probe set algorithms, and a single gene (myogenin) visualized for the time series (Figures 1, 2). The absolute expression calculations are shown (Figure 1; signal intensities = Y axis), and then the same data normalized to time 0 to generate a Y axis showing fold-change (Figure 2). MAS5.0, dCHIP difference model (Diff) [4], and Probe Profiler PCA (PCA) [5] correct for hybridization to the mismatch probes for this myogenin transcript, and lead to absolute expression levels near 0 at time 0 (Figure 1). On the other hand, RMA [6] and dCHIP perfect match only (PMonly) [7] do not correct for hybridization to the mismatch, and these show a very high expression of myogenin at time 0 (signals of 5,000–10,000) (Figure 1). From this difference, one can conclude that there was considerable hybridization to the mismatch probes, and that, at time 0, the mismatch and perfect match probe intensities were similar, leading to a level of "0 specific binding" for those probe set algorithms giving a penalty to the mismatch (MAS5.0, PCA, dCHIP Diff). On the other hand, the perfect match only probe set algorithms (dCHIP PMonly, RMA) make the assumption that much of the mismatch signal is "bona fide" signal from the myogenin transcript, and thus do not impart a penalty on the perfect match probes. This leads to very high signal for myogenin at time 0. While the "signals" are very different for myogenin between probe set algorithms, this is a difference in assumptions concerning background, not signal per se.

When the same data set is then normalized to time 0, the Y axis becomes a measure of "fold-change" between baseline (time 0), and peak expression (day 3) (Figure 2). As the graph now becomes a ratio of day 3 (numerator) and day 0 (denominator), the very different values for day 0 expression lead to very different ratios for the different probe set algorithms (Figure 2). In the example shown, the PCA probe set algorithm calculates a 90-fold increase in expression of myogenin (PCA), while the perfect match only probe set algorithms show only a 2-fold increase (RMA, dCHIP PMonly) (Figure 2). The volatility of the fold-change is a consequence of different calculations of the denominator for a ratio, and this is due to different assumptions concerning what is "background", and what is true "signal". If one takes this single example and extrapolates to all 40,000 probe sets on a microarray, one quickly understands why different probe set algorithms give very different interpretations of the same data.

## Comparison studies of probe set algorithms

There has been growing interest on the effect of probe set algorithms on data interpretation, with development of new probe set algorithms, and also comparisons between probe set algorithms (see [2] and [3] for reviews). A number of studies have found the lower variance of the perfect-match only algorithms, and other methods of reducing variance, to lead to more precision, with the conclusion that these provide the best performance [2,8-12]. However, one recent paper studying the issues of effects of normalizations methods on variance and data interpretation noted, "Any normalization reduces variance and increases sensitivity with no clear winner" [13]. Other authors have similarly cautioned against equating increased precision with improved performance, as precision may come at the cost of accuracy. The dramatic differences in fold-change interpretation of myogenin by different probe set algorithms shown in Figure 2 illustrate this point. The signal of the mismatch probes for myogenin is relatively high at time 0, but this mismatch signal does not rise proportionately with the increased myogenin perfect match signal seen at day 3. Thus, the greater precision of RMA and dCHIP PMonly comes at a cost of underestimating the fold-change at day 3. In other words, the balance of precision and accuracy would fall in favor of the greater accuracy of PCA, dCHIP diff, and MAS5.0 in this specific instance. Thus, appropriate assessments of "performance" may depend on a balance of precision and accuracy, and that this is likely project and probe set dependent [3,14,15].

An excellent R package to compare the performance of different probe set algorithms (Affycomp II) [16] has been used to compare over 50 probe set algorithms in a paper describing the effects on accuracy, precision, and sensitivity into low signals [2]. Consistent with expectations, the authors found that background correction (e.g. using mismatch signal) improved accuracy but worsened precision. They proposed that GC-RMA, PLIER, ZL, and RSVD algorithms provided the best balance of accuracy and precision [2]. This interpretation was based upon a spike-in data set, where a limited number of synthesized mRNAs were studied at varying concentrations.

We recently took a different approach, where three different large data sets with different biological variance intrinsic to each organism and experimental system (grass, rat, and human biopsy samples) were studied. We built an interactive analysis tool to query the statistical power of each probe set on the arrays in the project, with dynamic user-definition of sample size, effect size (fold-change), and statistical significance [3]. We found that the same algorithms that were found to provide excellent accuracy using spike in data [2], tended to reduce variance to inappropriate levels, leading to high false positives [3]. A recent paper found similar results where the majority of RMA-specific probe sets were unable to be validated by RT-PCR [8]. A generalization from all of these studies is that probe set algorithms that utilize only the perfect match probes give much better sensitivity with less variance (enhanced accuracy), particularly at low signal levels. However, these same low signals are particularly susceptible to strong effects from cross-hybridization from related mRNA sequences (non-specific hybridization), and other sources of undesired background signal. It should be kept in mind that in "real experiment" (e.g. not spike in data), the levels of both the desired (specific) transcript are changing, as well as the levels of the cross-hybridizing (non-specific) transcripts. This serves to make the system highly (if not impossibly) complex, and the ideal balance of precision and accuracy could be project and organism specific. Thus, all probe set algorithms should be viewed as a necessary but imperfect compromise in data interpretation.

## Is there a best bet?

One could envision that the ideal probe set algorithm would optimize background correction and normalization for each project and each probe set in that project. While such approaches are doomed to be "data starved", recently, new "hybrid" probe set algorithms have emerged that attempt to better combine the precision of model based approaches, with more judicious use of background correction (mismatch penalty). These include the Affymetrix PLIER algorithm [17] and GC-RMA [9]. The new hybrid algorithms have the potential to achieve a more ideal balance of accuracy and precision. To test this, we took a novel approach; namely statistical power analysis of "absent call only" probe sets in a microarray project. To explain this approach, the original MAS5.0 probe set algorithm looks at the signals from the 11 probe pairs in a probe set, and determines if the signals from perfect match probes exceed the signals from the paired mismatch probes. The statistical confidence that the ensembled perfect match signals from a probe set are significantly above background is derived as a "detection p value". If, on average, the perfect match probes show greater signal intensity than the corresponding mismatch, then the detection p value improves, to a threshold to where a "present call" is assigned (e.g. the target transcript is likely "present" in the sample tested). Whereas poor signal from the perfect match, and increasing signal from the mismatch, causes the detection p value to worsen, leading to an "absent call" determination. The "present call" is thus a statistically derived threshold reflecting a relative confidence that the desired mRNA is indeed present in the RNA sample being tested at a level significantly above background hybridization. An "absent call" suggests that the target mRNA is not present in the sample, or that there is non-target mRNAs binding to the probe set (non-desired cross-hybridization), or both. Thus, limiting data analyses to "present calls" can restrict analyses to the better performing probe sets and target mRNAs [18]. dCHIP algorithms also have an option to utilize the MAS5.0 "present calls" as a form of noise filter, and we have recently shown that use of the detection p value as a weighting function improves the performance of all probe set algorithms [19].

We reasoned that use of only those probe sets that showed 100% absent calls by MAS5.0 algorithms would enrich for poor quality signals with considerable noise components, due to poorly performing probe sets, low level or absent target mRNAs, or cross-hybridization. By limiting our statistical power analysis to these 100% absent call probe sets, we hypothesized that this would lead to a sensitive test for performance of probe set algorithms with regards to balance of precision and accuracy, and further, an assessment of the potential for false positives.

**Figure 3:**
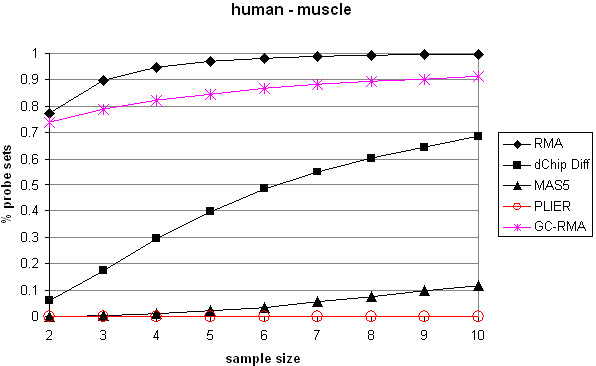
**Power calculations of absent call-only probe sets shows variable rates of false positives**. Shown is the output of HCE-power [20] for five probe set algorithms, including two newer "hybrid" algorithms (PLIER, GC-RMA), expressed as a % of probe sets fulfilling specific criteria (β = 0.2, α = 0.05, effect size = 1.5 fold change). Muscle biopsies from 16 normal controls, and 10 Duchenne muscular dystrophy patients were used on U133A microarrays [27], and the 8,200 probe sets that showed an "absent call" by MAS5.0 algorithm on all 26 arrays were then loaded into HCE-power. These "absent calls" reflect poorly performing probe sets, where there is low confidence that signal specific to the transcript is detected above background levels. By this analysis, both RMA and GC-RMA show significant powering of 70–80% of these "absent call" probe sets with only 2 microarrays per group. This can be interpreted as a high proportion of false positive results expected from this project using RMA or GC-RMA. On the other hand, PLIER shows insufficient powering for 98% of the 8,200 probe sets, even at group sizes of 10 arrays/group. This suggests that PLIER will show no false positives.

We used our public domain power analysis tool (HCE-power) [20] to study the effects on power analysis of two newer hybrid algorithms (PLIER, GC-RMA) relative to other popular algorithms (MAS5.0, RMA, and dCHIP difference model) (Figure 3). Power is the probability of calling a null hypothesis false when the null is actually false. By studying only poor signals (absent calls), many or most sufficiently powered probe sets will be prone to false positive results. Both RMA and GC-RMA showed statistically significant power for the large majority of these poorly performing probe sets, even at groups of n = 2. This suggests that both RMA and GC-RMA are subject to high false positive rates, consistent with conclusions of a recent study [3]. On the other hand, PLIER showed inability to appropriately power the large majority of probe sets, even at n = 10 per group (Figure 3). The exquisite precision of RMA and GC-RMA affords desired sensitivity into low signal ranges for these untrustworthy probe sets, but it is relatively unlikely that the derived signals correspond to specific hybridization. Thus, chip-to-chip fluctuations in background might lead to false positives from these probe sets when using RMA and also the GC-RMA hybrid algorithm. In the example shown, we can assume that the performance of the "absent call" probe sets is poor, with background signal and cross-hybridization to non-specific mRNAs leading to "signal" that is suspect. The data presented in Figure 3 suggests that improving precision through mathematical reduction of variance can become "inappropriate", and result in high risk for false positives. By this analysis, the PLIER algorithm appears the most stringent, with the lowest likelihood for false positives in poorly performing probe sets.

Another method of illustrating this point is to analyze sample mixing data sets. We used a publicly available dataset of two different human tissues (PBMC and placenta) mixed in defined ratios (100:0, 95:5, 75:25, 50:50, 25:75, 0:100) [21]. To enrich for signals near detection threshold, we selected all probe sets that were "present calls" in all 100% PBMC profiles, but absent calls in 75%, 50%, 25%, and 0% PBMC profiles. We then took all probe sets appropriately powered at n = 2 for PLIER and GC-RMA, and studies the ability of the two probe set algorithms to track dilutions of these low level PBMC-specific probe sets. Correlation analysis between PBMC RNA concentration and expression levels of the selected probe sets showed a statistically significant difference (F-Test p value = 1.43449E-05). As expected, GC-RMA (average R^2 ^= 0.244803) was considerably less accurate than PLIER (R^2 ^= 0.324908).

**Figure 4:**
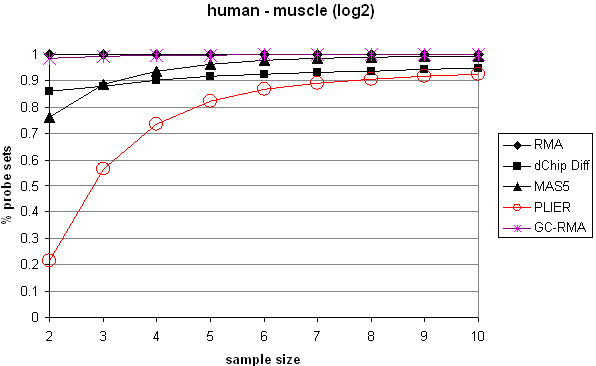
**Log transformation of absent call signals shows a strong reduction in variance, leading to a greater proportion of sufficiently powered probe sets through greater precision, but less accuracy (higher expected false positives)**. Log transformation of data is a commonly used method to reduce variance, and thus increase precision. Taking the same data set shown in Figure 3 and log transforming the data effectively increases the proportion of probe sets that are sufficiently powered at low numbers of arrays per group.

Log transformation of microarray data is another method that is frequently used to reduce variance, and hence improve precision [22-24]. We hypothesized that log transformation of the same data in Figure 3 would further mathematically reduce variance, improve precision and proportion of probe sets that are adequately powered, but with the disadvantage of leading to more potential false positives. Since the scale of signal values significantly influences power analysis results, we ran HCE-power with log2-scale data (Figure 4). Comparison of Figure 3 and Figure 4 confirmed our hypothesis: log transformation reduced variance and greatly increased the percentage of sufficiently powered probe sets (Figure 4). Interestingly, PLIER still showed the best performance in terms of the lowest potential false positive rates. This result suggests that the new dynamic weighting and error model for PLIER signal calculation is effective in reducing the influence of noise in the power calculation. It is important to note that the default signal calculations of the PLIER algorithm (obtained through R) include log transformation of the resulting signals. The data presented here suggests that log transformation of data should be used very judiciously; it may result in increased precision, but at a cost of accuracy. This interpretation is in agreement with other publications that have studied the balance of precision and accuracy [3,22].

## Conclusion

Efforts to use a single "best" probe set algorithm for all probe sets and projects may be an unattainable goal. Instead, selection of analysis methods for any particular project should be viewed as striving towards an appropriate balance of precision and accuracy. Emerging hybrid probe set algorithms, such as PLIER, may prove particularly powerful in reducing false positives. New tools such as Affycomp and HCE-power can be used to assess this balance.

## Authors' contributions

EPH conceived of the study. EPH and JS designed the study together. JS designed and implemented the power analysis tool (HCE-Power), and performed the statistical analysis. All authors read and approved the final manuscript.
